# Real-world vehicle emissions as measured by in situ analysis of exhaust plumes

**DOI:** 10.1007/s11356-017-9941-1

**Published:** 2017-08-23

**Authors:** Christian Peitzmeier, Carmen Loschke, Hanna Wiedenhaus, Otto Klemm

**Affiliations:** Institute of Landscape Ecology – Climatology, University of Münster, Heisenbergstr. 2, 48149 Munster, Germany

**Keywords:** Real-world vehicle emissions, Exhaust plume analysis, HBEFA, EU emission limits, Air quality in Europe, Diesel car emissions, Nitrogen oxides (NO_*x*_) emissions

## Abstract

We conducted a 60-day roadside measurement campaign on a busy street in Münster, Germany, during summer 2016. We used gas and particle concentration measurements with high temporal resolution (10 Hz) to quantify both the emission ratios of nitrogen oxides per carbon dioxide (NO_*x*_/CO_2_) for over 70,000 individual exhaust plumes as well as the emission ratios for size-resolved particle numbers per carbon dioxide (d(PN CO_2_^−1^)/dlogD) for about 10,000 plumes. The real-world fleet passing by the measurement station consisted of passenger cars (85%), buses (5.9%), light duty commercial vehicles (5.7%), trucks (1.7%), and motorcycles (1.6%). The median measured NO_*x*_/CO_2_ ratio was 3.33 g kg^−1^. The median measured PN/CO_2_ emission ratio for particles with diameters between 0.03 and 10 μm was 5.6 × 10^14^ kg^−1^. We compared our results with the Handbook Emission Factors for Road Transport (HBEFA) and the Euro 5 and Euro 6 emission standards by employing traffic counts, assuming the diesel-to-gasoline ratios of vehicles according to registration statistics, and estimating that stop-and-go traffic occurred 65% of the time. Using a conservative estimate, our median ratios exceeded the HBEFA data by more than 65% for NO_*x*_/CO and by a factor of about 100 for PN/CO_2._ Furthermore, our median NO_*x*_ emission per kilometer travelled (NO_*x*_ km^−1^) exceeded the Euro 5 emission limit for diesel cars by a factor of 3 and exceeded the Euro 6 limit by almost a factor of 7. Additionally, our median particle number emission (PN km^−1^) exceeded the Euro 5 and Euro 6 limits of diesel cars by a factor of almost 150. These results confirm the presumption that the emissions of a real-world traffic fleet comprehensively exceed the legal limits. Very likely, the widespread presence of defeat devices in vehicle emission control systems plays a major role in this discrepancy. This has a strong impact on the apparent inability of authorities to comply with the legal limits of the NO_2_ concentrations in urban air.

## Introduction

Air quality is a major concern in European cities, as major air pollutants such as nitrogen dioxide (NO_2_) and particulate matter are associated with several adverse health effects including cancer, respiratory, and cardiovascular diseases (Hoek et al. 2013; Elsaesser and Howard 2012). According to the European Environmental Agency (EEA 2016), 7% of the population of the European Union (EU) is exposed to concentrations of NO_2_ above the EU limits of 40 μg m^−3^ (yearly average), and 16% are exposed to excessive particle concentrations. Road transport is a main source of NO_2_ and also contributes to the ambient particle concentrations. In Germany in 2014, about 15% of the total particulate matter and 42% of nitrogen oxides (NO_*x*_ = NO + NO_2_) were estimated to originate from motor vehicles (UBA 2016a; UBA 2016b).

In order to prevent exposure to toxicologically relevant doses of air pollutants, the European Union established air quality standards (Directive 2008/50/EC 2008) as well as emissions standards for the type approval of light passenger and commercial vehicles (Regulation (EC) No 715/2007 2007). For the Air Quality Directive, the responsibility for clean air lies with the authorities managing the urban environment. Thus, local governments have put forth intense efforts to set conditions that lead to compliance with the limits, such as establishing no-entry zones in inner cities for cars that do not meet a given exhaust control level. On the other hand, regarding emissions standards, responsibility for ensuring that vehicles conform with the type approval regulations lies with vehicle manufacturers. While all these issues are interlinked, this study focusses on the emissions from individual street vehicles.

To this point, the type approval procedure for vehicles is performed on a roller dynamometer test bench in a clearly prescribed New European Driving Cycle (NEDC). This driving cycle is going to be replaced by the Worldwide Harmonized Light Vehicles Test Procedure (WLTP) in September 2017. This new driving cycle ensures a nationwide comparability of test procedures but includes no on-road tests (DELPHI 2017). In a different approach, real-world emissions from diesel cars and cars with spark-ignition engines are measured by Portable Emission Measurement Systems (PEMS) (Weiss et al. 2011; Kousoulidou et al. 2013; Ntziachristos et al. 2016). Several PEMS studies have shown that the Euro emission standards are frequently exceeded, especially the latest standard Euro 6 (Weiss et al. 2012; Franco et al. 2014; O’Driscoll et al. 2016). Almost all of the tested diesel vehicles exceeded the emission limits by at least a factor of 2 and up a factor of 22. This high discrepancy between in situ measurements and the test results within the NEDC for NO_*x*_ led the EU to develop a Real Driving Emissions (RDE) cycle, which is based on PEMS and random acceleration and deceleration on a public road at ambient conditions. Currently, the EU RDE cycle is employed just for observational purposes before it will come into force in September 2017 (European Commission 2015a, b; Commission Regulation (EU) 2016/646 2016; Commission Regulation (EU) 2016/427 2016).

The issue of high and harmful NO_2_ pollution in cities, as caused by road transport, became a worldwide media issue in September 2015. At that time, the US Environmental Protection Agency (EPA) uncovered a software in diesel car engines allowing them to cheat on emissions tests. These so-called defeat devices were installed in over 11 million cars produced by several car manufacturers including the German Volkswagen AG (VW) and its subsidiary companies (Yeomans 2015). The manipulated engine was introduced in Germany as early as summer 2007 and shortly thereafter in the USA. According to the Euro 6 regulation, the emission limit for passenger cars with diesel engine is 80 mg NO_*x*_ per kilometer in the European Union (Regulation (EC) No 715/2007 2007). In the USA, the Clean Air Act has set much stricter limits: manufacturers must ensure that their fleet averages NO_*x*_ emissions of 0.07 g mi^−1^ (43 mg km^−1^). Even stricter, the state of California allows an emission limit of only 0.05 g mi^−1^ (31 mg km^−1^; DELPHI 2017). In order to comply with these limits, engine exhaust needs to be cleaned with rather complex aftertreatment systems, which leads to a decrease in fuel economy and an increase of maintenance requirements. Thus, the software mentioned above was applied to disable the exhaust aftertreatment system whenever the cars were on the road but not when they were in the test stand. This so-called Dieselgate scandal is far from being concluded in Germany, Europe, or the USA (EPA 2016), and it is possible that the problem may be even more common. A recent study by Degraeuwe and Weiss (2017) demonstrated that Euro 4, Euro 5, and Euro 6 diesel cars produced by several manufacturers show substantially elevated NO_*x*_ emissions when driven on the road at conditions that are identical to those of NEDC type approval. These findings suggest that the application of defeat devices is widespread among manufacturers (Degraeuwe and Weiss 2017).

Exhaust-related particulate matter is currently less of an issue because air quality limits tend to be met at most stations, and, as far as is known, defeat devices only affect NO_*x*_ emissions but not particle emissions (UBA 2017). Yet, the Euro 6 emission standard restricts both particle mass (PM) and particle number (PN) emissions per kilometer in the type approval of diesel cars and gasoline cars with direct injection engines. As for NO_*x*_, the compliance of the emission limits for PM is also currently proven on a roller dynamometer test bench (Regulation (EC) No 715/2007 2007). The EU is planning to extend the RDE type approval procedure to PN emissions (European Commission 2016).

Given the mass confusion surrounding the actual emissions from cars, the scope of this project was to examine if exceedances of the emission limits for NO_*x*_, PM, and PN are a general phenomenon and, if so, how large these exceedances are in the real-world passenger car fleet within a town in Germany 2016. Thus, our objective was to measure real-world vehicle emissions at the roadside in order to calculate realistic emission factors for NO_*x*_, PM, and PN.

As mentioned above, on-board emission measurements using PEMS are appropriate methods to measure real-world emissions. However, their use is limited because they require elaborate measurement strategies and can therefore be applied only to a limited number of vehicles, making the upscaling of the results to a city atmosphere seemingly impossible. One promising way to acquire a representative sample of vehicles might be to use remote sensing techniques. Carslaw et al. (2011) set up an across-road vehicle emissions monitoring system (RSD-4600) in London based on measuring non-dispersive infrared (NDIR) and ultraviolet (UV) light across exhaust plumes. The measurement strategy was successfully realized for CO_2_, NO, NO_2_, and NO_*x*_, and further developments yielded reliable results for a large sample of single vehicles (> 70,000 vehicles) (Beevers et al. 2012; Carslaw and Rhys-Tyler 2013; Chen and Borken-Kleefeld 2014).

In our study, we used a different approach that can, in principle, be used for any component in vehicle exhausts for which a fast in situ sensing technique exists. We equipped a roadside container with continuously operating sensors for nitrogen oxides, carbon dioxide, ozone, and size-resolved particle concentrations. Fast sensing techniques allowed the identification and integration of peaks resulting from individual vehicles and groups of vehicles. Each plume was quantified in terms of its NO_*x*_/CO_2_ ratio and the ratios of particle size class concentration ratios versus CO_2_. Then, the results were compared to the data set provided by the Handbook of Emission Factors for Road Transport (HBEFA) (INFARS 2014) and the Euro 5 and Euro 6 emission standards, leading to conclusions concerning the ambient air quality and the fulfillment of European Air Quality Directives.

## Methodology

### Measurement site

We operated a measuring container at a two-lane road in the city center of Münster, Germany (51° 57′ 51.4″ N, 7° 37′ 47.2″ E) from May 3 to July 3, 2016. The road is an important east-west connection and within of the low-emission zone of Münster. The site is close to two crossroads and a major local bus station, which is served by 19 public bus lines (Stadtwerke Münster 2016a). Approximately 16,700 vehicles passed the measuring container per day.

The environmental protection agency of the federal state North Rhine-Westphalia (LANUV NRW) observes the NO_2_ concentration near the bus stop by using a passive sampler. The yearly means of NO_2_ were below 55 μg m^−3^ during the past 7 years with a continuously decreasing trend over the past 5 years (LANUV NRW 2016). The annual average NO_2_ concentration for the year 2016 was 43 μg m^−3^. An inductive-loop traffic detector has been in operation since June 1, 2016 and was located near the measuring container on the same road.

### Sensors

The measuring container was located on a pedestrian sidewalk at a distance of 4.5 m from the road. The sensors for CO_2_, temperature, humidity, and wind were installed at a height of 4 m above sidewalk level. The inlets of the intake tubes for the analytical sensors inside the container were also installed 4 m above sidewalk level. All sensors (Table 1) recorded at a frequency of 10 Hz.

**Table 1:** Employed sensors and their corresponding basic measuring principles to analyze traffic exhaust plumes

Sensor	Parameter	Measurement technique
ECO PHYSICS CLD 899 Y	NO, NO_*x*_	Chemiluminescence detection
ECO PHYSICS CLD 88	O_3_	Chemiluminescence detection
Licor Li7500A	CO_2_	Open path NDIR gas analyzer
ELPI+ (Electrical Low Pressure Impactor)	Particle number 14 size fractions	Unipolar corona charger + cascade impactor + electrometer

The ELPI 50% separation diameters are 0.006, 0.017, 0.030, 0.060, 0.108, 0.17, 0.26, 0.40, 0.64, 1.0, 1.6, 2.5, 4.4, 6.8, and 10 μm, respectively

The basic idea of our sensors setup is that each motor vehicle passing the container generates an exhaust plume, and it is reasonable to assume that the engine exhaust is well mixed with respect to gases and particles when exiting a vehicle’s muffler. Once emitted into the atmosphere, the plume will dilute with surrounding air masses. Therefore, the concentrations of typical exhaust gases such NO_*x*_ and CO_2_ will decrease. However, the concentration ratios of these gases will remain constant as long no significant reactions occur. For example, the concentration ratio of NO_*x*_ versus CO_2_ will remain constant at least within a time period of several minutes to hours, because CO_2_ does not react at all within this time period, and because the oxidation of NO_*x*_ to other gases such as HNO_3_ is too slow to significantly impact the NO_*x*_ concentration. For other gases, the respective concentration ratios within the plumes may not be considered to be constant within the time frame of a few minutes. This applies, for example, to the ratios NO/CO_2_ or NO_2_/CO_2_ due to the rapid reaction between NO, NO_2_, and O_3_.

Depending on the wind conditions, a vehicle exhaust plume may or may not reach the meteorological sensors and the inlets of our setup. For all plumes that reach the sensors and intakes, the air pollutants are detected as peaks of the CO_2_, NO_*x*_, and aerosol particles’ concentrations. For each plume, the increase of the concentrations of exhaust components over the respective background concentrations of urban air may be measured, and the ratios of the total increases can be quantified. This leads, for example, to the quantification of the ratio of NO_*x*_ versus CO_2_ in the vehicle exhaust. Note that it does not matter to what degree the plumes are diluted before detection as long as the increases in concentrations can be precisely measured. Whenever two or more individual plumes from single vehicles combine into a common peak, the peak may be analyzed as a whole.

An inductive-loop traffic detector was installed by the Münster city authorities within the roadway in immediate vicinity to the measuring container. Data was available for the entire month of June and at a temporal resolution of 15 min. As part of the counting system, vehicles were automatically classified into the classes of motorcycles, buses, trucks, and semi-trailer trucks (HDCV = heavy duty commercial vehicles), vans (LDCV = light duty commercial vehicles), cars, and cars with a trailer. We assumed that the traffic volume and fleet in June were representative for the entire measurement period.

Unfortunately, the induction loop does not provide any information about the fuel types of the counted vehicles, so the number of diesel vehicles needed to be estimated. Based on registration statistics for the federal state North Rhine-Westphalia (Kraftfahrt-Bundesamt 2016a), the relative share of diesel passenger cars to all passenger cars is 31.7%, and 94% of LDCVs are diesel vehicles. Additionally, buses and HDCV were considered to be exclusively diesel powered, while motorbikes have gasoline engines only.

### Peak detection and integration

The different measuring techniques of our sensors and the suction hoses caused a time shift of up to several seconds between the measured parameters. We assumed that each local maximum in the data set, i.e., each peak of the various gases and particle concentrations, resulted from a single exhaust plume or from a combined plume. The time shifts were quantified and eliminated by cross-correlation. All time series were referenced to the CO_2_ concentration. The time shifts were between 2.6 and 4.4 s.

**Fig. 1:**
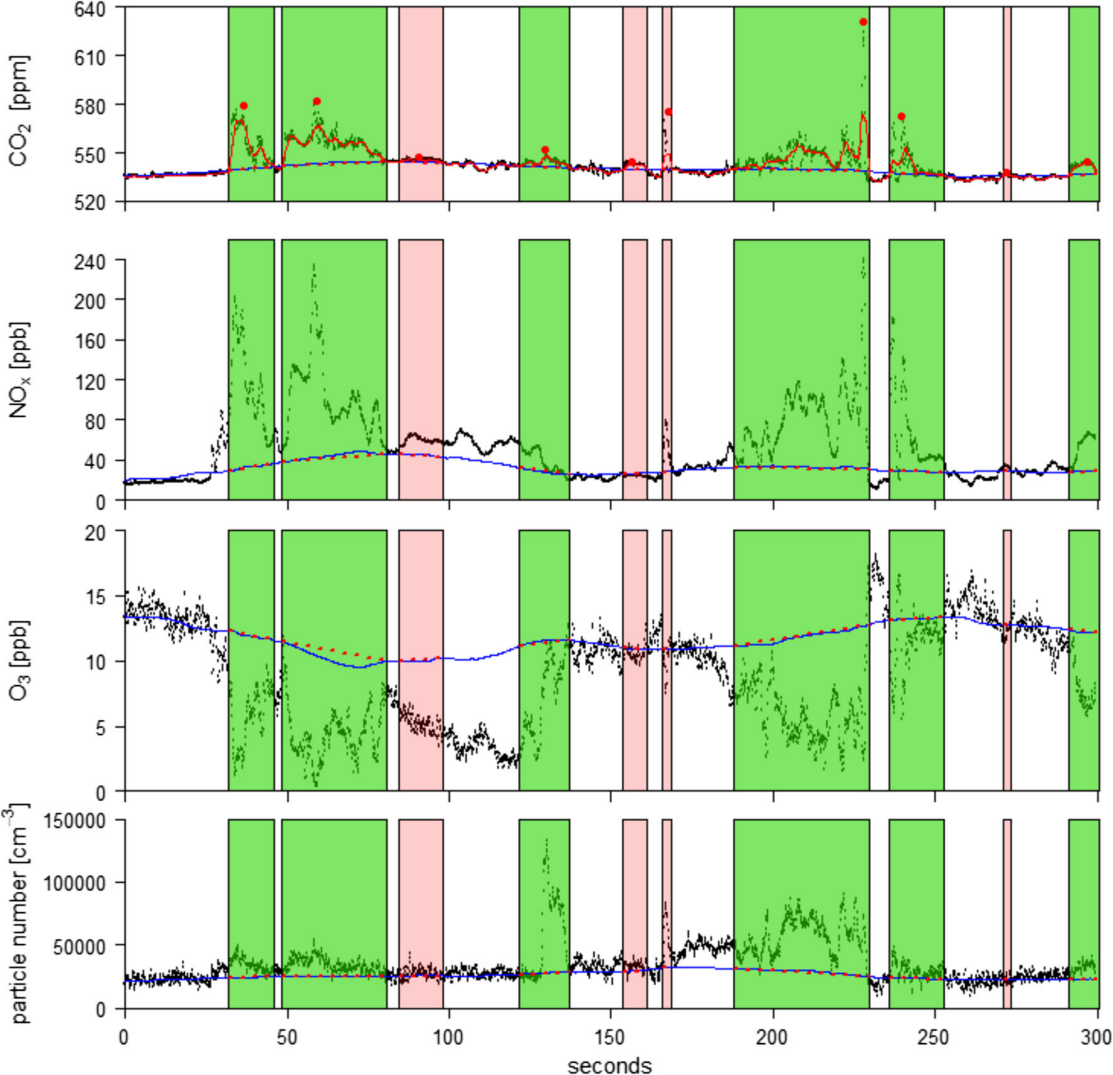
A 5-min section on May 21, 2016 from 09:10 to 09:15 h of the CO_2_ (ppm), NO_*x*_ (ppb), and O_3_ (ppb) mixing ratios and total particle number concentration (cm^−3^). All CO_2_ data points that have at least 100 neighboring data points with lower concentrations both upstream and downstream are marked as red dots. The red line in the CO_2_ time series is a moving average with a width of 25 data points. The PSL (blue line) and baseline (red dotted segments) were calculated individually for each substance. The green and pink rectangular areas mark identified peaks. The color indicates whether the peak passed (green) or failed (pink) the quality control. See text for more details.

We developed a semi-empirical method to detect and delimit the exhaust plumes in our data set. The identification process was based on the CO_2_ concentration. In a first step, local maxima (peaks) were defined as those points that had at least 100 neighboring data points with lower concentrations both upstream and downstream (Fig. 1, red dots). In the second step, the peak separation line (PSL, Fig. 1, blue line) was calculated. For this purpose, a moving average of 600 data points (1 min) and a moving standard deviation with a width of 6000 data points (10 min) were calculated. Data points that were more than half a standard deviation above the moving average were rejected and interpolated between the remaining data points within the last 10 min. The resulting data subset no longer contained local maxima (peaks). We used the de-spiked data set to calculate a moving average with 600 data points again to arrive at a trimmed CO_2_ background concentration, the PSL. All detected peaks from step 1 which were not above the PSL were rejected in the third step. In the fourth step, a further running average with a width of 25 data points was calculated directly from the CO_2_ concentration, including all peaks (Fig. 1, red line). This line, which represents the measured CO_2_ concentration rather well, frequently crosses the PSL, ideally two times in each interspace between two exhaust plumes. The closest intercept points before and after all remaining identified peaks from step 3 represented the beginning and end of an exhaust plume. If the end and beginning of two adjacent peaks were located at the same data point, the peaks were aggregated. The segment between the two intercept points forms the baseline of a peak integral (Fig. 1, red dotted segments). For peak integration, the integral of the peak itself (Fig. 1, between the data points and the red dotted segments) was considered while the sections below the red dotted segments were neglected; this procedure resulted in peak integrals. Peak separation lines were also calculated for the nitrogen oxides, ozone, and every particle size class. The integration procedure was analogous to that for CO_2_.

A final quality control was conducted before calculating the ratios between the peak integrals of the measured variables. Peaks with a duration shorter than 2 s were rejected, because the random error of the quantification of gas or particle amounts through peak integration was considered too large. Further, peaks longer than 150 s were rejected, because such long-wave structures can also be caused by the mixing in of air masses from suburban or rural spaces and could therefore not be clearly identified as locally produced exhaust plumes. If missing values (NA) occurred within the data points of a peak of one substance, the corresponding data points of all other substances were set to NA as well. If the ratio of these missing values was more than 25% of a peak’s duration, the exhaust plume was rejected and no further calculations were done. Furthermore, the absolute value of the sum of the values below the red dotted segments (negative values) was not allowed to be more than 25% of the sum of the positive values above the red dotted segments. On top of that, we set for each substance a minimum acceptable height of the peak’s maximum above the PSL. For this purpose, a moving standard deviation with a width of 6000 data points was computed, based on the same data set which was the basis for the calculation of the PSL. The median of all standard deviations for the whole measurement period is the substance-specific minimum height. Peak integrals that passed this quality control are shown in green in Fig. 1; peaks that did not pass the quality control are shown in pink.

Exhaust plumes of the particle data were only used for further analysis if the quality criteria were achieved in each of the 14 size fractions. The entire data analysis was performed with the software R (R Core Team 2016).

### Reference to HBEFA data and EU limits

The HBEFA provides emission factors for different vehicle types, pollutants, and traffic situations. We calculated average traffic-weighted emission factors based on these handbook emission factors and the measured traffic counts. For this purpose, the HBEFA software was operated for the conditions of a main road in an urban area with no slope, and a speed limit of 50 km h^−1^. Cold-start situations and systems like air conditioning were not taken into consideration. The results for all vehicle types were calculated for two traffic situations: low-emission fluid traffic and stop-and-go traffic (s-a-g).

To make our results comparable with HBEFA, the NO_*x*_/CO_2_ ratio was expressed in grams per kilogram. For each plume, the total particle number of each size class was referenced to the width of the size class and then referenced to the amount of CO_2_ in the respective exhaust plume. Assuming that the particles have a density of 1.3 g cm^−3^, the particle mass in each size class was calculated and referenced to CO_2_ as well.

The EU emission limits in the Euro 5 and Euro 6 type approval procedure of passenger cars for each pollutant refer to the distance travelled. In order to make our results (e.g.*,* in unit g kg^−1^ for NO_*x*_/CO_2_) comparable with these EU limits, we computed an average distance-specific CO_2_ emission of the passenger car fleet. For this purpose, we assumed that the traffic was always fluid and only passenger cars (diesel and gasoline-powered) passed the induction loop. Vehicles of other categories were omitted in this estimate because their emissions and emission limits are much more type specific, and there is no sufficient database about the vehicle fleet composition to make a more detailed analysis. Passenger cars are by far the biggest share in the total fleet. Based on these considerations and the HBEFA software, we computed an average traffic-weighted CO_2_ emission of 159 g km^−1^.

We calculated distance-specific emissions for NO_*x*_ from our data as

1 $$ \frac{\ {\mathrm{NO}}_x}{\mathrm{km}}\left[\frac{\mathrm{g}}{\mathrm{km}}\right]=\frac{{\mathrm{CO}}_2}{\mathrm{km}}\left[\frac{\mathrm{g}}{\mathrm{km}}\right]\times \frac{{\mathrm{NO}}_x}{{\mathrm{CO}}_2}\ \left[\frac{\mathrm{g}}{\mathrm{g}}\right] $$

The target value of NO_*x*_ emissions per kilometer (left-hand side of Eq. 1) is derived from the CO_2_ emission per kilometer as computed from HBEFA (159 g km^−1^) and the NO_*x*_/CO_2_ ratios for individual plumes from our measurements.

In order to compare our measured aerosol particle emission data with the EU emission limits for passenger cars, we used the same approach as for the NO_*x*_ emissions based on an average traffic-weighted CO_2_ emission of 159 g km^−1^. We omitted particles smaller than 23 nm in our results, which corresponds to the lowest two size fractions of the ELPI+, because the EU type approval procedure only restricts particles bigger than 23 nm in diameter.

## Results and discussion

During 60 days of measurement, the data availability was 84% for carbon dioxide, 93% for nitrogen oxides, and 79% for ozone. For the ELPI+ particle spectrometer, which had to be cleaned frequently, 38 days of data could be analyzed. A total number of 71,376 peak integrals (exhaust plumes) of CO_2_, NO_*x*_, NO, NO_2_, and O_3_ passed the quality control and were used for further calculation. For particle data, 9687 peak integrals could be used for further analysis.

### Traffic

**Fig. 2:**
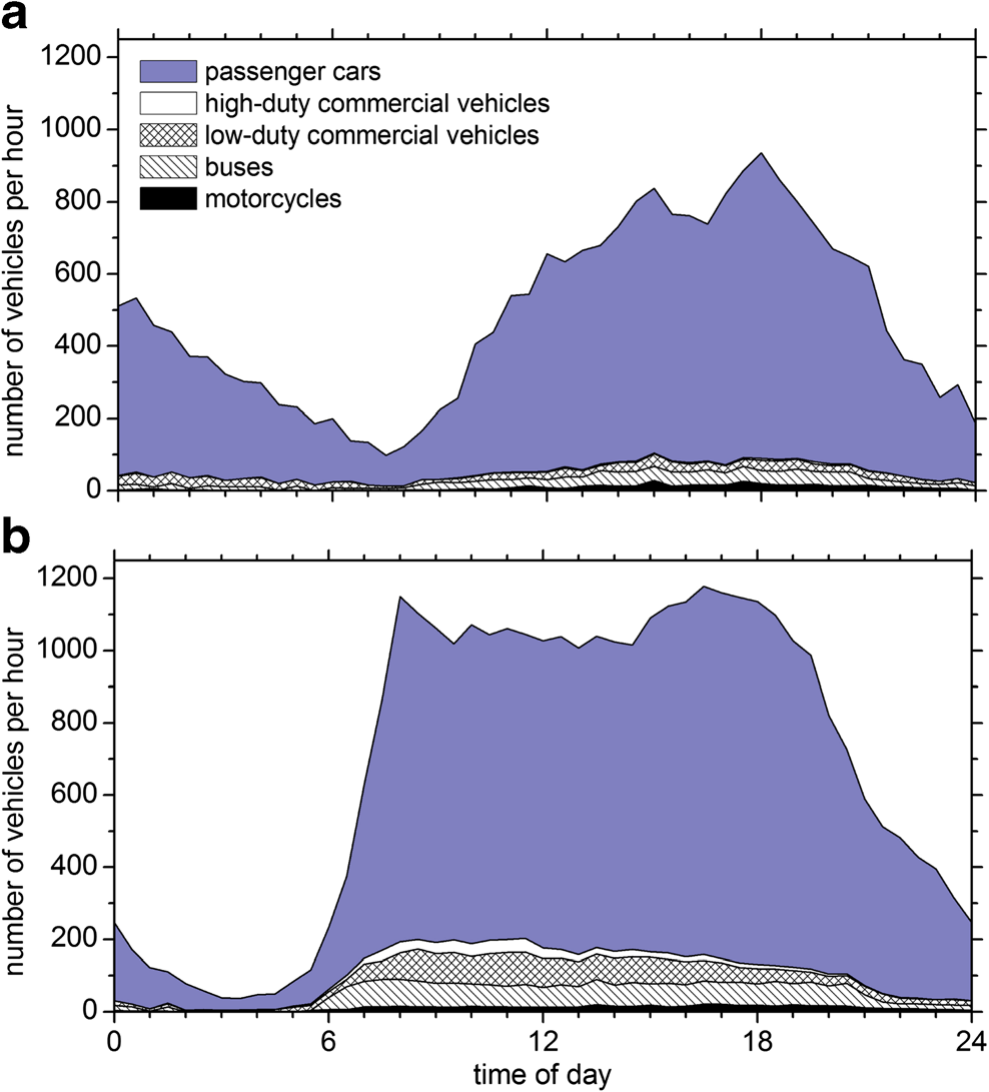
Median diurnal course of traffic counts, grouped into five vehicle classes. The upper panel (**a**) shows the data for Sundays; the lower panel (**b**) shows data from Mondays through Fridays. Heavy duty commercial vehicles (HDCV) include trucks, trucks with trailers, and semi-trailers. Light duty commercial vehicles (LDCV) are mostly vans. Cars with trailers were included in the cars class

On an average working day, the station was passed by about 16,700 motor vehicles, including 1000 buses, 1000 LDCV, and 330 HDCV. On Sundays, the total traffic was reduced by 30%, particularly the numbers of HDCV and LDCV were lower by about 60%. On every working day, the rush-hour traffic started at 06:00 h and increased from 230 to 1100 vehicles per hour until 08:00 h (Fig. 2b). The number of vehicles stayed above 1000 vehicles per hour throughout the day until 19:00 h. Then, the traffic volume dropped rather steadily through the night to reach a minimum of 50 vehicles per hour at 04:00 h. On Sundays (Fig. 2a), a traffic minimum of about 100 vehicles per hour occurred around 08:00 h, and then the traffic intensity rose to a maximum of 900 vehicles per hour by 18:30 h. A secondary maximum occurred during the night between 00:00 and 01:00 h. Overall, the site is characterized by a large number of public buses with two bus stops nearby, traffic lights, and by motor traffic that tends to be congested during the busy times of the working days. On Sundays and during the nights, the traffic flows rather unrestrictedly.

### Emission estimates from HBEFA

Average emission factors for each vehicle type are presented in Table 2. Several uncertainties arise from these data.

**Table 2:** Average emission factors for each vehicle type classified by fuel type and the traffic situations “fluid” and stop-and-go (“s-a-g”)

Vehicle type	Fuel	Number	Traffic situation	NO_*x*_/CO_2_ (g kg^-1^)	CO_2_ (g km^−1^)	NO_*x*_ (g km^-1^)	PN (N km^-1^)	PM (g km^-1^)
Motorbike	Gasoline	8674	fluid	1.1	8.2E+01	8.9E-02	−	−
			s-a-g	0.86	1.0E+02	8.8E-02	−	−
Bus/coach	Diesel	30,737	fluid	6.1	1.1E+03	6.4E+00	8.4E+13	9.1E−02
			s-a-g	9.1	1.8E+03	1.6E+01	1.7E+14	2.1E−01
LDCV	Gasoline	1700	fluid	1.3	1.7E+02	2.2E-01	8.7E+11	1.7E−03
			s-a-g	1.1	3.2E+02	3.5E-01	1.2E+12	1.9E−03
	Diesel	27,488	fluid	3.6	1.9E+02	6.7E-01	5.8E+13	3.7E−02
			s-a-g	3.1	3.0E+02	9.6E-01	1.2E+14	6.6E−02
Passenger car	Gasoline	304,924	fluid	0.48	1.6E+02	7.8E-02	6.3E+11	8.6E−04
			s-a-g	0.47	3.3E+02	1.5E-01	1.3E+12	2.0E−03
	Diesel	141,528	fluid	3.1	1.5E+02	4.7E-01	9.2E+12	9.1E−03
			s-a-g	3.3	2.8E+02	9.5E-01	1.9E+13	1.8E−02
HDCV	Diesel	8973	fluid	5.0	6.1E+02	3.1E+00	4.5E+13	4.1E−02
			s-a-g	6.8	1.2E+03	8.4E+00	1.1E+14	9.9E−02
Total fleet	All	524,024	fluid	1.8	2.2E+02	6.4E-01	1.2E+13	1.1E−02
			s-a-g	2.0	4.1E+02	1.5E+00	2.4E+13	2.4E−02

The calculation is based on the measured traffic count data in June 2016, registration statistics, and the emission factors by HBEFA (INFARS 2014)

First, the estimated share of diesel cars is rather uncertain. As the NO_*x*_ emissions of diesel-powered passenger cars are more than six times larger than those for gasoline cars (both as NO_*x*_/CO_2_ and as NO_*x*_/km), the uncertainty of the share of diesel cars leads to an uncertainty of the fleet’s emissions.

Second, both the inductive-loop traffic detector and HBEFA do not distinguish between coaches and urban public transport buses. Coaches typically emit much more pollutants than the very modern municipal public transport bus fleet, of which 60% meets the Euro 5 emission standard (Stadtwerke Münster 2016b). We used the mean values of these two bus types for our calculation (Table 2). Due to the frequently approaching buses at the nearby main bus stop, it is highly probable that the share of urban public transport buses is much larger than 50%. It can therefore be reasonably assumed that the calculated average emission factors for buses (Table 2), which were incorporated within the fleet’s emission estimates, are higher than they would be if a more detailed database was available. In this sense, our estimates (last two lines of Table 2) are conservative estimates in terms of our analysis. In other words, they are likely overestimates.

Thirdly, the HBEFA data is based on the exhaust analysis of only a limited number of vehicles. For example, the emission factors for Euro 6 cars are based on only 20 passenger cars covering 13 different models. Only one single gasoline-powered passenger car was tested. The diesel passenger cars, which were already on the market at the time of the measurement, are exclusively from the premium segment (Rexeis et al. 2013), and the analyses underlying HBEFA took place predominantly on roller dynamometers. This leads to rather large uncertainties of the emission factors of Euro 6 passenger cars under real-world driving conditions.

### NO_*x*_ emissions

#### NO_*x*_/CO_2_ ratios

**Fig. 3:**
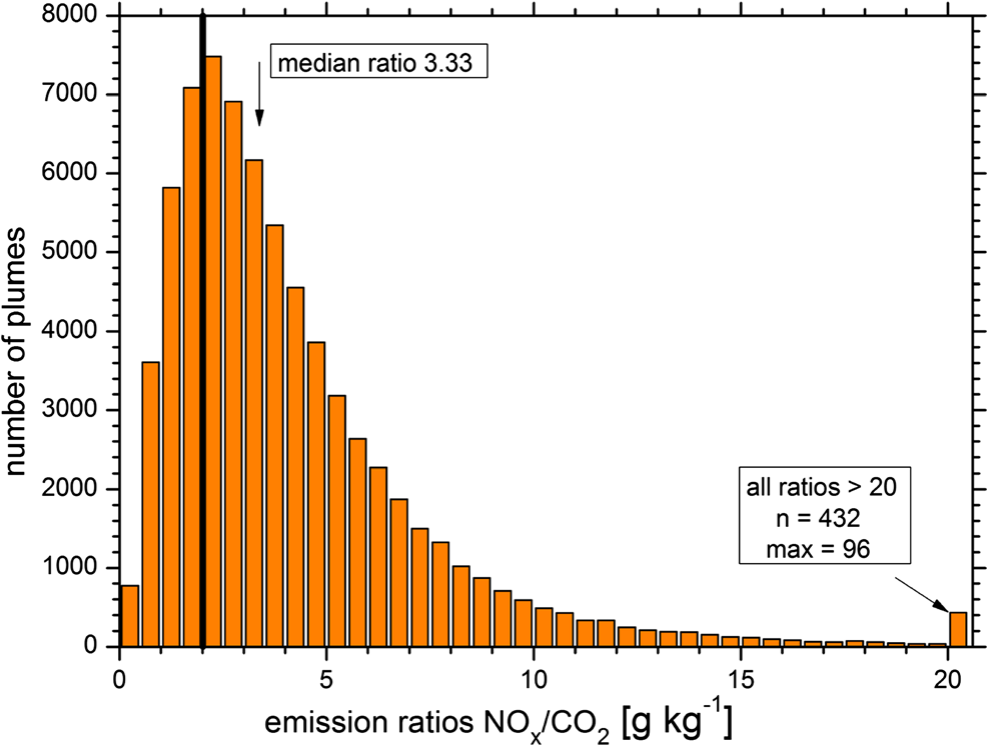
Frequency distribution of the measured NO_*x*_/CO_2_ ratios in kilograms per gram. The black line shows the calculated NO_*x*_/CO_2_ ratio based on the traffic count data and the emission factors according to HBEFA for stop-and-go traffic

The frequency distribution of the measured NO_*x*_/CO_2_ ratios in the exhaust plumes is shown in Fig. 3. The median NO_*x*_/CO_2_ ratio of all plumes is 3.31 g kg^−1^. The first and third quartiles are 2.04 g kg^−1^ and 5.28 kg g^−1^, and the 1 and 99% quantiles are 0.48 and 17 g kg^−1^, respectively. Overall, the median measured NO_*x*_/CO_2_ ratio is 65% larger than the NO_*x*_/CO_2_ ratio calculated from the emission factors according to HBEFA and traffic data for stop-and-go traffic (2.01 kg g^−1^; Table 2). Almost 75% of the exhaust plumes had a higher NO_*x*_/CO_2_ ratio than the ratio calculated from HBEFA. The uncertainties listed in the previous chapter provide no reasonable explanation for the large difference between our measured NO_*x*_ emissions and HBEFA. In fact, there are arguments indicating that the total NO_*x*_/CO_2_ ratio as estimated from HBEFA (Table 2, black line in Fig. 3) is a conservative estimate and is likely an overestimate. For example, there was obviously no persistent stop-and-go traffic during the entire measurement period; further, the share of coaches to total buses was most likely set too high. This strongly indicates that our estimated exceedances (75% of real-world NO_*x*_/CO_2_ emission ratios above HBEFA, median exceedance 65%) are rather large underestimates of the real exceedances.

Disagreements between the HBEFA database and real-world emissions were also detected by the abovementioned RSD measurement in London (Carslaw et al. 2011). In that study, the diesel car emissions were striking because they exceeded the HBEFA average over all Euro classes by more than 50%. The study by Chen and Borken-Kleefeld (2014) states that street emissions generally complied with the HBEFA emission factors when allowing a 6% engine deterioration per 50,000 km and accounting for engine load. Their study is based on the older HBEFA version 3.1, which may also contribute to differences between their results and ours. Uncertainties in emission models have also been detected by recent urban eddy covariance measurements in Innsbruck, Austria. Over a period of 1 year, the observed traffic-related NO_*x*_ emissions were 50 to 70% higher than those calculated with various up-to-date emission models (Karl et al. 2017). Overall, the widespread application of defeat devices is a probable cause for insufficient agreement between in situ measurements and models based on emission databases.

#### NO_*x*_ emissions in the context of EU limits

This section analyses the emissions of NO_*x*_ per distance travelled by individual vehicles. This leads to a comparison of our roadside measurements with Euro emission limits. We mainly refer to the latest Euro 5 and Euro 6 limits for passenger cars.

The median NO_*x*_ emission per kilometer, as derived from our plume analysis, exceeds the Euro 5 emission limit for diesel cars by a factor of 3 and those for gasoline cars by a factor of almost 9 (Table 3). The latest diesel emission standard (Euro 6) was exceeded by the median of our data by a factor of 6.6. The real-world results do not even comply with the oldest and highest limit for diesel car NO_*x*_ emissions (Euro 3 limit, 0.5 g NO_*x*_ km^−1^).

**Table 3:** Median measured emission factor for NO_*x*_ [g km^−1^], particle number, and particle mass in comparison with the Euro 1 to Euro 6 emission limits for gasoline and diesel passenger cars of class M1 < 2500 kg (Euro 1 to Euro 4) and class M (Euro 5 and Euro 6)

Emission factor	Fuel type	NO_*x*_ (g km^−1^)	PN (N km^−1^)	PM (g km^−1^)
Measured median		0.526	8.95E+13	4.54E+00
Euro 1 limits	Diesel	−	−	1.40E−01
	Gasoline	−	−	−
Euro 2 limits	Diesel	−	−	8.00E−02
	Gasoline	−	−	−
Euro 3 limits	Diesel	0.5	−	5.00E−02
	Gasoline	0.15	−	−
Euro 4 limits	Diesel	0.25	−	2.50E−02
	Gasoline	0.08	−	−
Euro 5 limits	Diesel	0.180	6.00E+11	4.50E−03
	Gasoline	0.060	−	5.00E−03^a^
Euro 6 limits	Diesel	0.080	6.00E+11	4.50E−03
	Gasoline	0.060	6.00E+12^a^	4.50E−03^a^

^a^Particulate mass and number limits apply only to vehicles with direct injection engines

Certainly, vehicles other than Euro 5 and Euro 6 passenger cars passed our site. If we would have been able to take vehicles into account, the average CO_2_ emission per kilometer would have been substantially higher with the consequence that the calculated NO_*x*_ emission per kilometer would have been much higher as well. With these arguments taken into consideration, our NO_*x*_ emission factors per kilometer would exceed the EU emission limits even further.

High exceedances of the latest Euro 5 and Euro 6 limits by diesel passenger cars in real-world operation were also detected in several other studies. Franco et al. (2014) tested 15 Euro 6 diesel passenger cars in a PEMS study over a driving distance of 6400 km. The NO_*x*_ emissions per kilometer were, on average over all analyzed cars, seven times higher than the respective Euro 6 limit. These results are representative across testing regions, manufacturers, and aftertreatment technologies.

A recent overview of Anenberg et al. (2017) confirms that in all major vehicle markets, the NO_*x*_ emissions of diesel vehicles do not comply with respective emission limits. For Euro 3 to Euro 5 light duty vehicles, the NO_*x*_ emissions are consistently high, on the order of 0.8 g km^−1^, although EU limits were tightened step-by-step from Euro 3 to Euro 5. For Euro 6 vehicles, the emissions are lower. However, this needs to be compared with the strictly tightened emission limit of Euro 6 in comparison to Euro 5 (Table 3). The relative exceedance of the (improved) emissions in Euro 6 light duty vehicles over the strictly tightened Euro 6 emission standards is not less than it is for the earlier Euro classes.

### Aerosol emissions

#### Particle number/CO_2_ ratio and particle mass/CO_2_ ratios

**Fig. 4:**
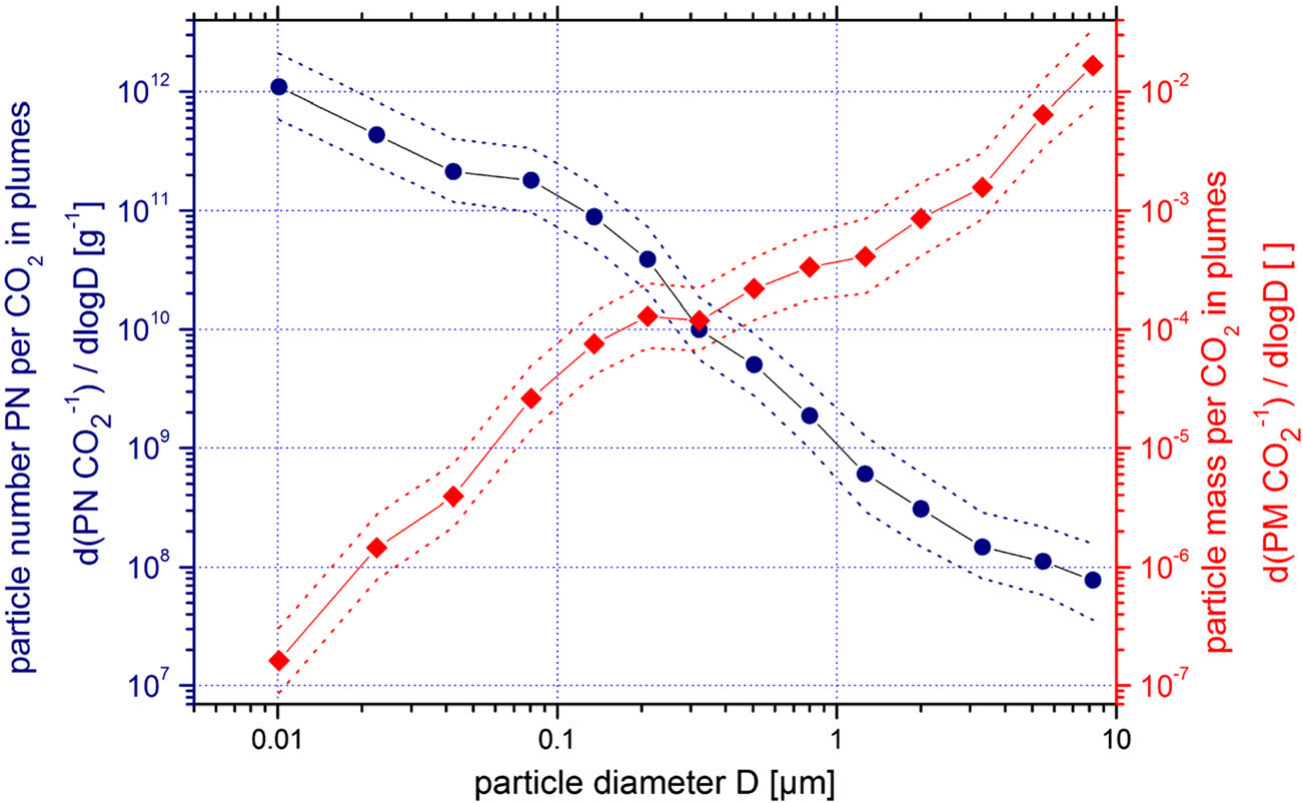
Size spectrum of the median ratio of particle numbers (PN) (see Table 1) per mass CO_2_ within each particle size channel in all analyzed plumes in units of per gram, including the first and third quartile (left *y*-axis, blue lines and dots). Size spectrum of the median ratio of particle mass (PM) per mass CO_2_ within each particle size channel in all analyzed plumes in units of grams per kilogram, including the first and third quartile (right *y*-axis, red lines and squares)

The particle numbers and particle masses of each size fraction were also referenced to CO_2_ in the respective exhaust plumes. A size-segregated analysis was performed. The total particle number concentration (Fig. 4, blue lines) is strongly dominated by ultrafine particles (diameter < 0.1 μm). In a median exhaust plume, 90% of the particles have an aerodynamic diameter smaller than 0.1 μm. On the other hand, the particle mass size distribution (Fig. 4, red lines) is dominated by coarse particles. Particles with an aerodynamic diameter larger than 1 μm constitute about 96% of the particle mass in a median exhaust plume.

The particle mass concentrations in plumes cannot be exclusively assigned to the engine exhausts; the particle mass in plumes is also affected by non-exhaust particles such as abrasion from tires and brakes as well as resuspension and swirling of particles from the road surface as caused by the passing traffic (UBA 2009). Unfortunately, we cannot distinguish between these different particle sources in our data set.

#### Aerosol emissions in the context of EU limits

There is no comparable data set in HBEFA setting limits to size-segregated aerosol particle number emissions. Nevertheless, when summing up the classes which are comparable to the HBEFA particle size range (diameters > 23 nm), our emissions are higher than those of HBEFA for Euro 5 and Euro 6 diesel cars by a factor of 150 (Table 3) and those for gasoline cars with direct injection engines by a factor of 15. Unfortunately, there is no estimate for cars older than Euro 5 and no HBEFA regulations either for further comparison. Only about 9% of the car fleet that passed our site fulfills the Euro 6 norm.

## Conclusion

We made in situ analyses of vehicle exhaust plumes at a busy urban street in order to quantify the emission ratios of trace gases and aerosol particles. We analyzed over 70,000 individual plumes, offering a rather representative sample of real-world emissions, i.e., the actual traffic fleet passing by. Depending on the prevailing meteorological conditions, only a fraction of the actual emission plumes was transported to the intake tubes of our roadside sampling site. Some plumes reached the station quicker, i.e., within a few seconds, while others likely took longer to become detectable. Also, the exhaust plumes of several vehicles may have been combined into one single plume before detection. All these boundary conditions do not have a negative effect on the experimental setup itself as long as no appreciable chemical reaction took place that might alter the detected emission ratios. We conclude that our concept yields valid data.

Due to the large number of plumes analyzed, our method yields data that are representative for the total vehicle fleet that passed our site. We consider it to be easily transferable to other seasons, other settings, and operation conditions, as long as individual plumes can be individually detected and analyzed. The application to shorter experimental periods with much lower numbers of plumes analyzed will lead to a larger statistical uncertainty, which will have to be quantified in such cases. The methodology is, in principle, extendable to any other gaseous or particulate compound of interest for which a fast-enough analytical technique is available. This might apply to a suite of organic compounds detectable by mass spectrometry.

We developed an R software package to identify and quantify peaks in the time series which are interpreted as vehicle plumes. Background concentrations were disregarded through the introduction of dynamic concentration baselines. The routine worked well and is transferable to other situations, although the parameterizations defining the peak detection may have to be adapted for different settings in the urban environment. And, while it is virtually impossible to estimate the uncertainty of individual plume analyses, we presume that there is no source of any systematic error. The random errors for individual plumes should be in the range of the uncertainty of the analytical instruments (few %) but should be much smaller for larger ensembles such as those evaluated here.

In order to compare our results to current standards on vehicle emissions such as the HBEFA database or the Euro 5 and Euro 6 emission limits for road transport, the data sets needed to be transformed into common units such as emission ratios of grams per kilogram between two gases, or emission rates per distance travelled, in grams per kilometer. This leads to rather large uncertainties because no detailed statistics are available about the vehicle fleet passing our measurement site. The traffic counts only classify the vehicles by weight and length, but no information about the age of cars, their type of engine (diesel or gasoline), and emission standard (Euro 1 through Euro 6) is available. We derived estimates using simplifying assumptions such as statewide registration statistics. All our estimates are conservative in the sense that stated exceedances of emission databases and EU emission limits are most likely understatements.

The NO_*x*_/CO_2_ emission ratios varied widely in our data set, from 0.48 to 17 g kg^−1^ (1 to 99% range). The median of 3.3 g kg^−1^ was 65% higher than the estimated HBEFA average of 0.20 g kg^−1^ for our traffic fleet. Most of the individual plumes (almost 75%) were above this value. Translating this data into NO_*x*_ emission rates per distance travelled, our fleet exceeded the EU emission limits by factors of 3 to 9. This is a significant exceedance, even upon the background of rather large uncertainties in the fleet’s emission data. This result is in general agreement with most other studies of NO_*x*_ emissions from vehicles. Various studies follow various experimental strategies. For example, PEMS studies focus on individual cars and driving conditions, while our study was designed to measure as many vehicle emission plumes as possible in order to reflect the real-world conditions as well as possible. It is rather striking that studies employing very different experimental approaches arrive at very similar results.

Given the fact that the annual average NO_2_ concentration at the nearby operational air quality site was 43 μg m^−3^ for 2016 and thus exceeded the EU limit value by 3 μg m^−3^, our results imply significant consequences for the efforts to comply with the EU regulations. Assuming an urban background NO_2_ concentration of 22 μg m^−3^ (indicators -lanuv.nrw.de), another 21 μg m^−3^ of NO_2_ (annual average) results from the local traffic. If the contribution to local traffic could be reduced by the same amount that the median NO_*x*_ emissions were found to exceed the median HBEFA emission in this study (165%), this would yield a traffic contribution of NO_2_ of only 13 μg m^−3^. When this value is combined with background NO_2_, this would give a hypothetical yearly average NO_2_ concentration of only 34 μg m^−3^, which is below the EU air quality limit of 40 μg m^−3^. Although this rough estimate is associated with large uncertainties, for example, those concerning the reaction of NO with O_3_ in exhaust plumes, the respective calculations (not shown in detail) on the basis of the exceedance of emissions per distance travelled (in units g km^−1^) would lead to even further reductions of the annual NO_2_ concentration. We are therefore confident that compliance of vehicle emissions with the legal regulations would avoid the exceedance of the air quality standard of NO_2_ at our street site in Münster.

For aerosols, the exceedance of our measured emission uncertainties is larger than for NO_*x*_. For the total particle numbers of diameters > 23 nm, the existing Euro 5 and Euro 6 emission limit per distance travelled is exceeded by a factor of 150. It is difficult to evaluate the representativeness of the result since no emission limits for vehicles older than Euro 5 exist. For the particle mass, the emissions per kilometer travelled derived from our plume analysis is even larger (factor of 1000); however, this result should not be overrated, because much of this particle material may have consisted of a resuspension of particles from the street surface. Nevertheless, the particle numbers and masses in exhaust plumes were impressive. Further research should be done to investigate how effective operational particle filters are in real-world traffic.

Our analysis clearly suffers from the fact that no more detailed information about the traffic passing our measuring site was available. Further experiments should therefore include a license plate recording with subsequent inquiry of vehicle information from the federal administration. This can largely reduce the uncertainties associated with this type of study. Further, a second measurement campaign during the cold season should be made. Low-exchange weather conditions, high relative humidity, and low ambient air temperatures may affect the particle number concentration (Yao et al. 2007) and size distribution. Separate identification of resuspended material should also be performed.
